# Clinical Characteristics of Youth with Autism or Developmental Disability during Inpatient Psychiatric Admission

**DOI:** 10.3390/jcm11216328

**Published:** 2022-10-27

**Authors:** Emily Neuhaus, Anthony Osuna, Daina M Tagavi, Sina Shah-Hosseini, Shannon Simmons, Jennifer Gerdts, Alysha D Thompson

**Affiliations:** 1 Department of Psychiatry & Behavioral Sciences, University of Washington, Seattle, WA 98195, USA; 2 Seattle Children’s, Seattle, WA 98145, USA; 3 Department of Psychology, University of Washington, Seattle, WA 98195, USA

**Keywords:** autism spectrum disorder, developmental disability, inpatient psychiatry, psychiatric hospitalization, internalizing, externalizing, medication, psychiatric comorbidity, equity

## Abstract

Children with autism spectrum disorder and developmental disabilities (ASD/DD) often experience severe co-occurring psychological and behavioral challenges, which can warrant inpatient psychiatric care. However, very little is known about the characteristics and clinical care of children with ASD/DD within the context of inpatient psychiatric settings. In this paper, we describe factors unique to inpatients with ASD or DD, by drawing on electronic health records from over 2300 children and adolescents ages 4–17 years admitted to a pediatric psychiatric inpatient unit over a 3-year period. Patients with ASD/DD accounted for approximately 16% of inpatients and 21% of admissions, were younger, more likely to be readmitted, more likely to be male, and more likely to have Medicaid insurance, as compared to patients without ASD/DD. Clinically, those with ASD/DD more frequently had externalizing concerns documented in their records, in contrast to more frequent internalizing concerns among other patients. Within the ASD/DD group, we identified effects of patient age, sex, and race/ethnicity on multiple dimensions of clinical care, including length of stay, use of physical restraint, and patterns of medication use. Results suggest the need for psychiatric screening tools that are appropriate for ASD/DD populations, and intentional integration of anti-racist practices into inpatient care, particularly with regard to use of physical restraint among youth.

## 1. Introduction

Individuals with autism spectrum disorder and those with intellectual disability, developmental differences due to known genetic events (e.g., Klinefelter’s), or other developmental disability (hereafter referred to as ASD/DD) face unique challenges that put them at elevated risk for significant psychiatric conditions. It is estimated that around 70% of autistic youth have at least one co-occurring mental health condition and about 40% have two, with substantial variability across estimates [1,2]. More broadly, the co-occurrence rate of mental health disorders among youth with ASD/DD has been found to be as high as four to five times greater than the rate among typically developing youth [3]. A recent meta-analysis by Lai and colleagues [4] estimated the prevalence of mental health concerns within autistic samples, identifying high rates of ADHD (28%), anxiety disorders (20%), sleep-wake disorders (13%), disruptive behavior disorders (12%), depressive disorders (11%), obsessive compulsive disorders (9%), bipolar disorders (5%), and schizophrenia spectrum disorders (4%).

Considering these high rates of co-occurring mental health conditions, many individuals with ASD/DD are likely to require high levels of psychiatric care [4]. However, individuals with ASD/DD tend to have more limited access to mental health care compared to typically developing children [5,6]. As a result of this limited access to outpatient services in conjunction with high acuity of mental health challenges, a substantial proportion of youth with ASD/DD require higher levels of mental health care, including inpatient psychiatric settings. ASD/DD youth are admitted to psychiatric hospitals at disproportionately high rates, with an estimated 11% of autistic youth needing inpatient hospitalization before the age of 21 [7].

Righi and colleagues [8] examined variables that predicted inpatient psychiatric hospitalization among autistic youth aged 4–20 years across six specialized inpatient units (Autism Inpatient Collection [AIC]) [9]. Results indicated that lower adaptive functioning, greater severity of ASD social communication symptoms, having a single primary caregiver, sleep problems, and the presence of a mood disorder were all independent predictors of increased risk for psychiatric hospitalization. Other predictors in autistic youth include aggressive and self-injurious behavior and psychiatric comorbidities [10]. Horowitz and colleagues [11] identified that 22% of an autistic inpatient sample experienced high levels of suicidal ideation. While these studies provide insight into which children may ultimately require this level of psychiatric care, there is still much to understand about the characteristics and experiences of youth with ASD/DD admitted for inpatient care. Those most severely affected by ASD/DD, including those with expressive language impairment, low adaptive functioning, and/or externalizing behaviors have been especially understudied [12].

Within the inpatient setting, overlap between features of ASD/DD and psychiatric disorders may complicate both diagnostic understanding and identification of appropriate interventions to support the complex needs of patients. Clinicians may encounter the challenge of diagnostic overshadowing, the tendency to over-attribute a patient’s symptoms to a particular condition while overlooking a comorbid condition [13], making accurate conceptualization difficult. Furthermore, some youth with ASD/DD may have limited insight into their emotional experiences [14,15] as well as limited communication skills, which may interfere with self-report of internal symptoms important to differential diagnosis.

Inaccurate or incomplete diagnostic understanding may hamper the development and implementation of appropriate treatment plans and may both limit the effectiveness of inpatient care and expose youth to disproportionate risk. Among autistic youth included in the AIC, over 90% received at least one psychotropic medication during admission [16], in contrast to rates among outpatient samples of approximately 55–65% [17]. Although psychotropic medication is evidence-based and potentially helpful for many conditions and symptoms in youth with ASD, medication also brings potential risks that may compound with prolonged use (e.g., [18]). Similarly, use of physical restraint in psychiatric settings is common among patients with externalizing behaviors and self-injurious behavior [19], but carries physical and psychological risks for patients and staff [19]. Such findings underscore the importance of accurately identifying psychiatric features among youth with ASD/DD to inform treatment approach and minimize unintended consequences.

In recognition of such barriers to understanding psychiatric disorders within youth with ASD/DD, admission to inpatient psychiatric facilities offers a unique opportunity to examine the characteristics and experiences of this especially vulnerable population. The current paper describes data from a large, single-site study of youth admitted to an inpatient psychiatric unit over the course of a three-year span. Our objectives were threefold: (1) to consider demographic features among inpatient youth with and without ASD/DD, (2) to characterize rates of psychiatric features within the sample, and (3) to explore associations between demographic and clinical care characteristics among youth with ASD/DD and those without. Better understanding of inpatient experiences will help us identify and address health disparities and improve services for the ASD/DD population.

## 2. Materials and Methods

### 2.1. Participants

Data were extracted from electronic health records (EHRs; EPIC and CIS) for children and adolescents between the ages of 4 and 17 years who were admitted to a psychiatric inpatient unit, called the Psychiatry and Behavioral Medicine Unit (PBMU), within a large, regional pediatric academic medical facility. All patients in the current dataset completed their admission in the 3-year time frame from 2019 through 2021 (inclusive; timeframe selected due to EHR availability). There were no exclusion criteria with regard to clinical presentation or demographic factors. This yielded a sample of 2385 unique patients, who accounted for a total of 3612 admissions completed during the timeframe of interest. Within this full sample, 16.2% of patients were identified through EHRs as having autism, intellectual disability, developmental differences due to known genetic events (e.g., Klinefelter’s), and/or an unspecified developmental disability. We refer to this group as the ASD/DD group, while patients without those features documented in the EHR are referred to as the non-ASD/DD group. See Table 1.

### 2.2. Procedures

For all patients in the sample, we extracted a number of variables from the EHR using Tableau software. Demographic variables of interest included patient age (in whole years), sex assigned at birth, race and ethnicity, and insurance under which inpatient care was received. Approximately 40% of patients had personal pronouns designated in their EHR, whereas the remaining 60% were admitted prior to standardized documentation of pronouns. When possible, a proxy variable to index gender identity was developed by identifying patients for whom specified personal pronouns differed from those associated with their sex assigned at birth. See Table 1 for demographic features of the sample.

Variables related to a range of psychiatric features were also extracted from patients’ EHR “problem list” in order to characterize the sample clinically. Individual clinical features (e.g., panic, agoraphobia) were grouped into larger categories (e.g., anxiety disorders) informed by DSM-5 diagnostic groupings [20], and each category yielded a variable indicating presence or absence of that clinical feature for each patient. In addition to diagnostic features, clinically relevant factors such as exposure to adverse childhood experiences (e.g., death of family member, family disruption) and history of trauma (e.g., maltreatment) were included. Note that features were not mutually exclusive, and our approach allowed patients to have multiple clinical features endorsed if documented in their EHR. See Table 2 for the list of features included in the current data set.

In addition, we extracted variables indicating characteristics of clinical care during each admission. These included length of admission, number of admissions during the 3-year timeframe of interest, use of physical restraint during admission, and use of pro re nata (PRN) or “as needed” medications for anxiety/aggression, sleep, and pain. Note that medication data were not available for the full 3-year timeframe so analyses with medication variables pertain to a subset of that time.

### 2.3. Analytic Approach

Our first goal was to better understand the characteristics of patients with ASD/DD in the inpatient setting. T-tests (for continuous variables) and chi-squares (for categorical variables) were computed to compare patients with ASD/DD versus those without on demographic, psychiatric, and clinical care characteristics.

Next, we examined associations between demographic and clinical care characteristics within the ASD/DD and non-ASD/DD subgroups separately. Demographic factors of interest in these analyses included patient age in years (as a continuous variable), sex assigned at birth (male, female), race/ethnicity, and insurance status (Medicaid vs. private/self-pay). Note that for these analyses, our variable for race and ethnicity was adapted to include categories with at least 5% of the sample, in order to maintain the de-identified nature of data and preserve confidentiality for patients in smaller groups. This approach yielded the following self-reported groups: Black or African American, Hispanic, non-Hispanic white, and two or more races. A fifth group included patients who reported their race as American Indian or Alaska Native, Asian, Native Hawaiian or other Pacific Islander, or other, as well as patients for whom race was not reported. Gender identity was omitted as a predictor in these models given that it was not available for the full sample.

Clinical care outcomes of interest for these models included (1) length of admission, (2) number of admissions occurring within our 3-year timeframe, (3) use of physical restraint, (4) use of PRN medication for anxiety or aggression, (5) use of PRN medication for sleep, and (6) use of PRN medication for pain. For restraint and medication use, variables included both a dichotomous indicator (presence/absence of use) during each admission as well as a continuous indicator for those admissions in which use was present (average instances per day for restraint, number of instances per admission for medication use). Across these variables, logistic regression models were used to consider presence/absence of clinical features (e.g., use of restraint), with linear regression models to consider number of instances among those with at least one instance. Given the limited literature on these factors among youth with ASD/DD, we followed an exploratory approach in our analyses and applied *p*-values of 0.05 for all analyses.

## 3. Results

### 3.1. Demographic and Psychiatric Features among Patients with and without ASD/DD

A total of 2385 unique patients completed admissions on the PBMU during the 3-year period considered here. Demographic information is presented in Table 1. Patients ranged in age from 4 years to 17 years, with a mean age for the full patient group of 13.47 years. Over half (60.2%) were assigned female at birth, 39.7% were assigned male at birth, and 0.1% declined to provide sex assigned at birth. Approximately 40% of participants had personal pronouns documented in the electronic health record; of those, 41.5% indicated pronouns other than those matching sex assigned at birth.

Within the full patient group, 16.2% of patients had ASD, ID, or developmental disability indicated as a diagnosis within their electronic health record, substantially higher than rates in the general population [21]. Compared to patients without those diagnoses, those with ASD/DD were younger at the age of admission, more frequently assigned male at birth, less likely to have documented pronouns that differed from their sex assigned at birth, and less likely to receive care through commercial insurance. Patients with ASD/DD did not differ from those without in the distribution of self-reported race and ethnicity. See Table 1.

Next, we explored the frequency with which a series of psychiatric features were documented for patients with and without ASD/DD. For those with multiple admissions within our 3-year time period, we considered only the patient’s first admission. See Table 2 for frequencies and group comparisons.

As shown, the most frequently documented psychiatric features for the full sample were those associated with internalizing disorders, with depressive features documented among approximately two thirds of the sample (67.3%), and both suicidal ideation (54.7%) and anxiety (56.4%) noted for over one half of the sample. Externalizing features were also noted, but with lesser frequency.

Analyses comparing groups, however, indicated substantial differences in the frequencies of psychiatric features according to the presence of ASD/DD. For patients without ASD/DD, frequencies mirrored those in the full sample, such that features related to depression, suicidal ideation, and anxiety were documented for the majority (74.2%, 59.7%, and 59.6%, respectively). Group comparisons indicated that these internalizing features were noted significantly more often for patients without ASD/DD than for those with ASD/DD. Features related to substance use, trauma and stress, feeding and eating, and self-injury were also documented more frequently among patients without ASD/DD.

Conversely, for patients with ASD/DD, the most frequently documented psychiatric features were those related to externalizing disorders: attention-deficit and hyperactivity (74.6%), and disruptive, impulse-related, and conduct problems (64.8%). In contrast to internalizing features, these externalizing features were documented significantly more frequently among patients with ASD/DD than among those without.

We next examined characteristics of inpatient admissions for the full sample and by group, as shown in Table 3.

Together, this cohort of patients accounted for a total of 3612 admissions over the 3-year timeframe of interest. On average, patients had a mean length of admission of approximately 10 days, and patients with ASD/DD did not differ from those without in this regard. However, on average, patients with ASD/DD had significantly more inpatient admissions with the 3-year study period.

Clinical care characteristics documented during inpatient admissions also differed between those with and without ASD/DD. The use of physical restraint was more likely among those patients with ASD/DD, with nearly a quarter (24.3%) of that group experiencing at least one instance of restraint during their inpatient stay, in contrast to 13.8% of admissions for patients without ASD/DD. Of those admission in which restraint was used, though, the mean number of instances per day did not differ across groups.

Patterns of PRN or “as needed” medication use differed according to group and medication target. With regard to medication for aggression or anxiety, medication was used in a greater percentage of admissions and more frequently per admission for patients with ASD/DD. A similar pattern was observed for medication addressing sleep, which was again used in a greater percentage of admissions and more frequently per admission for our ASD/DD group.

In contrast to these medications, PRN medication use to address pain did not differ significantly in the percentage of admissions or in the number of instances per admission across the two groups.

### 3.2. Associations between Demographic and Clinical Factors within the ASD/DD Subgroup

Our second objective was to explore associations between demographic and clinical factors in order to better understand how these factors may interact. Logistic and linear regression model results are presented in Table 4 for patients with ASD/DD and in Table 5 for patients without ASD/DD.

*Length of admission*. Among patients with ASD/DD, age and race/ethnicity significantly predicted length of admission such that length of stay was longer for participants who were older. With regard to race, contrasts indicated that Black or African American patients had significantly longer stays on average than white patients (15.26 versus 9.74 days, respectively). Among patients without ASD/DD, length of stay was again longer for older patients.

*Use of physical restraint*. Patient age and race/ethnicity also predicted use of restraint among our group with ASD/DD. Use of restraint was more likely for younger patients (OR = 0.92, 95% CI [0.87–0.98]) than for older patients. Black or African American patients were nearly three times as likely to experience restraint than non-Hispanic white patients (OR = 2.74, 95% CI [1.56–4.81]).

Among admissions that included at least one instance of restraint, instances were more common among female patients than male patients (0.55 versus 0.28 instances per day, respectively). Insurance status was also significant, with more instances of restraint per day for patients who received care through Medicaid versus private insurance or self-pay (0.46 versus 0.28 instances per day, respectively).

For patients without ASD/DD, restraint was again more likely when patients were younger (OR = 0.911, 95% CI [0.88–0.95]), and also when they were male (OR = 1.32, 95% CI [1.05–1.66]) and had Medicaid insurance (OR = 1.42, 95% CI [1.13–1.77]). The effect of race was significant as well, with higher likelihoods of physical restraint for patients who were Black or African American (OR = 1.99, 95% CI [1.35–2.91]) and for those reporting race that was less than 5% of the sample (OR = 1.35, 95% CI [1.01–1.81]), relative to non-Hispanic white patients.

Among those who did experience physical restraint, instances were significantly less frequent among Hispanic patients than among non-Hispanic white patients (0.21 versus 0.39 instances/day, respectively).

*Use of PRN medication for anxiety or aggression*. For patients with ASD/DD, the model testing likelihood of receiving medication for anxiety or aggression was not significant. However, among those with ASD/DD with at least one instance of medication use, instances were more frequent for patients who were older. A marginal effect of race and ethnicity was also observed, with more frequent medication use for patients who endorsed two or more races, relative to non-Hispanic white patients (31.5 versus 18.72 instances, respectively).

For patients without ASD/DD, use was more likely for patients who were older at the time of admission (OR = 1.16, 95% CI [1.10–1.23]). In the subset who received PRN medication, instances were more frequent for those who were older, male (15.50 versus 12.73 instances, respectively), and had Medicaid insurance (14.82 versus 12.45 instances, respectively).

*Use of PRN medication for sleep*. Among patients with ASD/DD, models exploring PRN use of medication for sleep were not significant with regard to likelihood of use. Among patients with at least one instance of PRN medication for sleep, instances were more frequent for patients who were older, and those who endorsed two or more races, relative to non-Hispanic white patients (39.35 versus 20.29 instances, respectively).

Patients without ASD/DD were more likely to receive PRN medication for sleep if they were older (OR = 1.08, 95% CI [1.02–1.15]), and if they had Medicaid insurance (OR = 1.65, 95% CI [1.15–2.37]). Among those who received medication at least once, use was more frequent for patients who were older and those with Medicaid insurance (15.30 versus 13.51 instances, respectively).

*Use of PRN medication for pain*. For patients with ASD/DD, likelihood of PRN use of medication to address pain was associated with age and sex. Use of pain medication was more likely when patients were older (OR = 1.11, 95% CI [1.03–1.20]) and when they were female (OR = 0.48, 95% CI [0.29–0.79]). The subsequent model exploring frequency of medication use among patients with at least one instance did not reach significance.

For patients without ASD/DD, likelihood of PRN medication for pain was higher for older patients and females (OR = 0.40, 95% CI [0.29–0.54]), with a marginal effect of race and ethnicity that did not reach significance. Among patients who received PRN pain medication, instances were more frequent among older patients.

## 4. Discussion

In this paper, we identify a number of features unique to the characteristics and experiences of youth with autism and developmental disabilities during inpatient psychiatric hospitalization. Most fundamentally, youth with ASD/DD were overrepresented on the inpatient unit and were significantly more likely to be readmitted, accounting for 16.2% of patients and 20.7% of admissions. The nature of our inpatient unit-the only unit in the region to provide inpatient psychiatric care for youth with severe ASD/DD-likely accounts in part for this elevation. However, these figures far exceed community-based prevalence of autism in the general population (estimated to be between 0.7 and 2.3%; [21,22,23]), indicative of considerably heightened risk for hospitalization among this group.

Together, these findings underscore the significant mental health needs of this population, and highlight the need for more accessible outpatient mental health services tailored to ASD/DD, both to pre-empt the need for inpatient care and to reduce the likelihood of readmission through better implementation of continuous care following discharge [24]. Although inpatient psychiatric care can elicit very meaningful improvements in behavior and mental health [25,26], hospitalization also removes a child or teen from their established support networks (e.g., school, therapists, childcare providers), disrupts routines and structure on which they may depend for self-care and other daily tasks, and introduces unfamiliar sensory demands (e.g., available food options, ambient lighting and noise) [24]. While best practice recommendations include evaluating factors such as sensory needs and communication supports to develop appropriate accommodations for each individual [27], not all youth have access to inpatient units with ASD-informed care, and efforts to decrease the need for hospitalization are important. Likelihood of inpatient admission may be decreased through access to ASD-appropriate outpatient mental health services [24], but also by respite care [28] and consistent access to other forms of therapy (speech, occupational) that are frequently recommended for youth with ASD/DD [24]. Data suggest these protective effects are observable over the course of months, and thus these services must be accessible, consistent, and established well before a mental health crisis.

### 4.1. Implications

Our data reveal a number of differences in documented psychiatric features among youth with and without ASD/DD. Those with ASD/DD had higher rates of externalizing features relative to peers, but lower rates of depression, anxiety, and suicidal ideation and attempts. This contrasts with outpatient data indicating heightened internalizing disorders in ASD (e.g., [4]), as well as with elevated rates of suicidal ideation and behavior among adults with ASD [29,30].

These differences suggest that internalizing features may be overlooked or misattributed among patients with ASD, perhaps due to patients’ difficulties with emotional insight and self-report, or a tendency toward diagnostic overshadowing among clinicians. Clinicians may also have difficulty recognizing internalizing symptoms that are expressed behaviorally. For example, a child with a developmental disability who engages in aggressive behavior in response to severe anxiety, panic, or trauma may receive an externalizing diagnosis that fails to capture their underlying anxiety symptoms, particularly in the context of limited verbal communication skills [31]. This possibility is bolstered by the number and variety of diagnostic features that were documented more frequently among patients without ASD/DD in our sample, and further suggests a missed opportunity for more nuanced and thorough conceptualization and documentation of psychiatric features for patients with ASD/DD. Similar considerations emerge with regard to PRN use of psychotropic medications in this sample. Patients with ASD/DD were more likely to receive medication to address anxiety, aggression, or sleep than non-ASD/DD patients. However, this pattern did not extend to medication for pain, which could suggest misattribution of pain symptoms that manifest as anxiety, aggression, or sleep disturbances.

These possibilities reinforce the need for nuanced diagnostic conceptualization to inform appropriate treatment. One step toward such understanding is the implementation of standardized, routine screening of psychiatric features across symptom domains for youth with ASD/DD, specifically using tools that have been validated or developed among this population. Such tools are currently rare, but continued research will aid in developing novel measures that accurately characterize mental health in the context of ASD/DD (e.g., Emotion Dysregulation Inventory) [32], adapting existing measures to better fit youth with ASD/DD (e.g., Anxiety Scale for Children-ASD; [33]), and evaluating the validity of established pediatric mental health measures among youth with ASD/DD (e.g., [34,35]).

Most concerning among our findings is the markedly increased rates of physical restraint among patients who were Black or African American, compared to those who were white. Existing research has demonstrated increased likelihood of restraint among Black youth during inpatient psychiatric admissions [36], and also among youth with developmental diagnoses [19,37]. Black or African American youth with ASD/DD experience the intersection of these identities, culminating in a threefold likelihood of physical restraint during inpatient psychiatric care in the data presented here. As a result, they likely disproportionately experience the detrimental sequelae of physical restraint in the form of trauma-related responses and physical injury [38], compounded atop the psychiatric crisis motivating their inpatient stay.

These findings call for intervention from multiple approaches concurrently, including anti-bias training for clinicians and systemic shifts away from physical restraint practices. Bias within mental health care impedes access, quality, and patients’ experiences of care [39]. Programs aiming to reduce implicit bias have been implemented in some settings, although evidence of improved patient outcomes is lacking [40]. A number of programs also exist as alternatives to restraint, with growing evidence of effectiveness in reducing instances of physical restraint and increasing patients’ satisfaction and sense of safety [38,41]. These include Trauma-Informed Care, which integrates restraint prevention (e.g., verbal de-escalation), trauma-informed principles, data-informed practices, staff debriefing, and other strategies [38,41]. Programs specific to autism and intellectual disability also exist (e.g., Autism Spectrum Disorder Care Pathway [42]) and decrease restraint through components including structured/predictable schedules, coping skills, and communication support.

### 4.2. Limitations and Future Directions

Extraction of clinical data from patient EHR in this manner has advantages with regard to sample size and inclusion, but does not distinguish diagnostic groups (e.g., ASD, intellectual disability) within the broad category of developmental disabilities. Similarly, this approach does not permit evaluation of individual differences in cognitive ability, language skills, or other phenotypic characteristics that likely influence psychiatric presentation and inpatient experiences [43]. As one example, our data indicate that, among patients with ASD/DD who experienced physical restraint, females had significantly more instances than males. Possible contributors to this difference could be variability in the severity of aggressive or self-injurious behaviors prompting restraint, differences in receptive and expressive language skills needed for alternative de-escalation strategies, or differences in patients’ history of behavioral therapy. Our data cannot speak to these possibilities, but exploration of individual differences in these features, as well as broader family and environmental factors (e.g., caregiver mental health, family structure, access to health care), will be important moving forward to describe mental health trajectories and inform supports for youth with ASD/DD.

Furthermore, data in this paper cannot speak to the experiences of youth with ASD or DD who had not yet been identified at the time of inpatient admission. With an age range of 4 to 17 years, our sample likely includes children or adolescents for whom an ASD diagnosis is warranted but not yet considered or evaluated, particularly if features of ASD have been obscured by the presence of co-occurring psychiatric symptoms (e.g., [44]). This population of later-diagnosed individuals are at particular risk for suicidal ideation and death by suicide [30], and warrant attention in an inpatient setting where such concerns are prevalent. We cannot determine the extent which our findings extend to that subset of youth, and it may be that they differ with regard to psychiatric features (e.g., higher rates of internalizing symptoms, lower rates of externalizing symptoms) and characteristics of care.

Finally, because our data come from a single site rather than a network or consortium (e.g., AIC), factors specific to our institution and/or region may influence our results and limit generalizability to other sites. Related, our data include admissions both prior to the emergence of COVID-19 and during the pandemic, and the effects of the pandemic and associated hospital or regional policies (e.g., school closures, social distancing restrictions) on the outcomes and factors of interest in this study are not clear. One possible effect of lockdown conditions might be increased psychiatric acuity for admissions overall, as systems that would otherwise have supported children and teens were unavailable. For example, youth likely encountered difficulty accessing outpatient mental health supports due to school and agency closures, while their caregivers likely experienced increased caregiving, financial and employment stress [45]. Once admitted, difficulty identifying appropriate post-discharge mental and behavioral health services may have necessitated longer admissions and increased re-admissions. While speculative, these possibilities will be worth exploring in future research.

Looking ahead, continued research into the experiences of children and adolescents with developmental disabilities including autism is essential in order to provide evidence-based psychiatric care within inpatient and outpatient settings. Two essential steps in this work will be the incorporation of first-hand perspectives from individuals with autism or developmental disabilities, and further exploration of the inpatient experiences of youth with ASD/DD who are members of marginalized groups. Through these methods, we aim to better understand characteristics, improve equity, and increase the effectiveness of inpatient care for children and adolescents with autism and developmental disabilities.

## Figures and Tables

**Table 1:** Demographic information for PBMU patients with and without ASD/DD during 3-year study timeframe.

	All	ASD/DD	Non-ASD/DD	Group Comparisons
*N* = 2385 Unique Patient		16.2% of Patients	83.8% of Patients	
***Age in year***				*t* = 8.56, *p* < 0.001
*Mean (SD)*	13.47 (2.76)	12.39 (3.23)	13.68 (2.61)	
*Range*	4–17	4–17	5–17	
***Sex assigned at birth***				*Χ*^2^(1) = 240.66, *p* < 0.001
*Female*	60.2%	24.9%	67.0%	
*Male*	39.7%	75.1%	32.9%	
***Pronouns relative to sex assigned at birth***				*Χ*^2^(2) = 21.36, *p* < 0.001
*Differ*	16.5%	8.5%	18.0%	
*Match*	23.3%	26.4%	22.7%	
*Unknown*	60.2%	65.0%	59.3%	
***Race & ethnicity***				*Χ*^2^(8) = 7.29, *p* = 0.506
*American Indian & Alaska Native*	1.6%	1.3%	1.7%	
*Asian*	4.5%	3.90%	4.7%	
*Black or African American*	6.1%	6.70%	6.0%	
*Hispanic*	13.5%	10.6%	14.1%	
*Native Hawaiian & Pacific Islander*	0.5%	0.3%	0.6%	
*Non-Hispanic White*	56.7%	61.1%	55.8%	
*Two or more races*	6.9%	6.0%	7.1%	
*Unknown or Declined*	6.4%	7.0%	6.3%	
*Other*	3.8%	3.1%	3.9%	
***Insurance***				*Χ*^2^(2) = 6.41, *p* = 0.04
*Commercial*	57.0%	51.3%	58.1%	
*Medicaid*	40.4%	45.3%	39.5%	
*Self-pay or other*	2.6%	3.4%	2.5%	

**Table 2:** Psychiatric features documented among PBMU patients with and without ASD/DD.

	All	ASD/DD	Non-ASD/DD	Group Comparisons
*n* = 2385 Unique Patients		16.2% of Patients	83.8% of Patients	
***More frequent in ASD/DD group***				
*Disruptive, impulse control, or conduct disorders*	25.6%	64.8%	18.0%	*Χ*^2^(1) = 371.59, *p* < 0.001
*Attention deficit & hyperactivity disorders*	35.2%	74.6%	27.6%	*Χ*^2^(1) = 314.05, *p* < 0.001
*Homicidal ideation or violent behavior*	8.5%	13.0%	7.7%	*Χ*^2^(1) = 11.67, *p* < 0.001
***No significant difference by group***				
*Obsessive compulsive and related disorders*	4.4%	4.7%	4.4%	*Χ*^2^(1) = 0.07, *p* = 0.79
*Schizophrenia spectrum and related disorders*	5.4%	4.9%	5.5%	*Χ*^2^(1) = 0.18, *p* = 0.67
*Bipolar and related disorders*	3.1%	1.8%	3.3%	*Χ*^2^(1) = 2.42, *p* = 0.12
*Trauma history*	15.1%	12.4%	15.6%	*Χ*^2^(1) = 2.54, *p* = 0.11
*Exposure to stress and adverse childhood experiences*	10.5%	7.8%	11.1%	*Χ*^2^(1) = 3.70, *p* = 0.054
***More frequent in non-ASD/DD group***				
*Self-injury*	27.0%	18.7%	28.7%	*Χ*^2^(1) = 16.44, *p* < 0.001
*Feeding & eating disorders*	8.1%	2.3%	9.2%	*Χ*^2^(1) = 20.35, *p* < 0.001
*Suicidal attempt*	9.5%	3.1%	10.8%	*Χ*^2^(1) = 21.97, *p* < 0.001
*Trauma and stressor related disorders*	21.8%	11.9%	23.8%	*Χ*^2^(1) = 26.59, *p* < 0.001
*Substance use and abuse*	9.6%	2.1%	11.1%	*Χ*^2^(1) = 30.30, *p* < 0.001
*Anxiety disorders*	56.4%	39.9%	59.6%	*Χ*^2^(1) = 50.97, *p* < 0.001
*Suicidal ideation*	54.7%	29.0%	59.7%	*Χ*^2^(1) = 122.78, *p* < 0.001
*Depressive disorders*	67.3%	31.1%	74.2%	*Χ*^2^(1) = 273.51, *p* < 0.001

Notes. The following features were not analyzed due to low incidence (<3%) in the cohort: catatonia, dissociative disorders, personality disorders, somatic disorders, tic disorders.

**Table 3:** Characteristics of clinical care during inpatient admissions among patients with and without ASD/DD.

	All	ASD/DD	Non-ASD/DD	Group Comparisons
*n* = 3612 Admissions		20.7%	79.3%	
***Length of admission in days***				
*Mean (SD)*	10.08 (12.14)	10.70 (12.93)	9.91 (11.92)	*t* = −1.57, *p* = 0.116
*Range*	0–253.5	0.1–230.6	0–253.5	
***Admissions per patient within 3-year study period***				
*Mean (SD)*	1.51 (1.16)	1.81 (1.68)	1.46 (1.02)	*t* = −5.47, *p* < 0.001
*Range*	1–14	1–14	1–11	
***Use of physical restraint***				
*% of admissions*	16.0%	24.3%	13.8%	*Χ*^2^(1) = 48.12, *p* < 0.001
Mean per day *	0.36 (0.42)	0.37 (0.52)	0.35 (0.37)	*t* = −0.55, *p* = 0.58
Range *	0.01–4.74	0.01–4.74	0.01–2.22	
Use of PRN medication for aggression or anxiety **				
*% of admissions*	68.8%	82.1%	64.6%	*Χ*^2^(1) = 33.18, *p* < 0.001
Mean per admission *	15.23 (16.31)	19.94 (18.27)	13.38 (15.09)	*t* = −5.49, *p* < 0.001
Range *	1–98	1–98	1–96	
Use of PRN medication for sleep **				
*% of admissions*	83.2%	90.9%	80.7%	*Χ*^2^(1) = 17.15, *p* < 0.001
Mean per admission *	16.32 (16.86)	22.27 (20.18)	14.24 (15.00)	*t* = −6.99, *p* < 0.001
Range *	1–99	1–99	1–97	
Use of PRN medication for pain **				
*% of admissions*	48.5%	44.3%	49.7%	*Χ*^2^(1) = 2.78, *p* = 0.095
Mean per admission *	4.76 (7.44)	5.28 (9.26)	4.62 (6.86)	*t* = −0.92, *p* = 0.36
Range *	1–86	1–65	1–86	

Notes. * indicates that mean values and ranges include only admissions where event of interest occurred at least once. ** indicates that medication data were available for a subset of encounters (1294 encounters).

**Table 4:** Models evaluating demographic factors and clinical outcomes for patients with ASD/DD.

	Overall Model	Age	Sex Assigned at Birth	Race and Ethnicity	Insurance
***Length of Admission (Days)***					
Mean per admission *	***F*(7,738) = 3.08,** ***p* = 0.003**	***F*(1,738) = 4.76,** ***p* = 0.029**	*F*(1,738) = 2.58, *p* = 0.108	***F*(4,738) = 2.40,** ***p* = 0.049**	*F*(1,738) = 1.08, *p* = 0.299
***Use of physical restraint***					
*Presence/absence*	**χ^2^(7) = 19.94,** ***p* = 0.006**	***B* = −0.08,** ***p* = 0.005**	*B* = −0.28, *p* = 0.15	***Wald* = 12.38,** ***p* = 0.015**	*B* = −0.05, *p* = 0.799
Mean per day *	***F*(7,173) = 3.66,** ***p* = 0.001**	*F*(1,173) = 0.12, *p* = 0.727	***F*(1,173) = 12.20,** ***p* < 0.001**	*F*(4,173) = 1.46, *p* = 0.216	***F*(1,173) = 9.52,** ***p* = 0.002**
Use of PRN medication for aggression or anxiety **					
*Presence/absence*	χ^2^(7) = 4.13, *p* = 0.765	**--**	--	--	--
Mean per admission *	***F*(7,248) = 2.74,** ***p* = 0.009**	***F*(1,248) = 8.38,** ***p* = 0.004**	*F*(1,248) = 1.53, *p* = 0.217	*F*(4,248) = 2.34, *p* = 0.056	*F*(1,248) = 0.00, *p* = 0.971
Use of PRN medication for sleep **					
*Presence/absence*	χ^2^(7) = 9.46, *p* = 0.222	**--**	--	--	**--**
Mean per admission *	***F*(7,276) = 3.59,** ***p* = 0.001**	***F*(1,276) = 7.61,** ***p* = 0.006**	*F*(1,276) = 2.13, *p* = 0.146	***F*(4,276) = 3.66,** ***p* = 0.006**	*F*(1,276) = 0.00, *p* = 0.968
Use of PRN medication for pain **					
*Presence/absence*	**χ^2^(7) = 24.20,** ***p* = 0.001**	***B* = 0.10,** ***p* = 0.009**	***B* = −0.74,** ***p* = 0.004**	*Wald* = 5.61, *p* = 0.23	*B* = 0.35, *p* = 0.16
Mean per admission *	*F*(7,131) = 0.95, *p* = 0.47	**--**	--	--	--

Notes. * indicates that analysis includes only admission where event of interest occurred at least once. ** indicates that medication data were available for a subset of encounters (1294 encounters).

**Table 5:** Models evaluating demographic factors and clinical outcomes for patients without ASD/DD.

	Overall Model	Age	Sex Assigned at Birth	Race and Ethnicity	Insurance
***Length of Admission (Days)***					
Mean per admission *	***F*(7,2851) = 2.34,** ***p* = 0.022**	***F*(1,2851) = 14.39,** ***p* < 0.001**	*F*(1,2851) = 0.00, *p* = 0.976	*F*(4,2851) = 0.29, *p* = 0.887	*F*(1,2851) = 0.02, *p* = 0.897
***Use of physical restraint***					
*Presence/absence*	**χ^2^(7) = 67.48,** ***p* < 0.001**	***B* = −0.09,** ***p* < 0.001**	***B* = 0.28,** ***p* = 0.016**	***Wald* = 13.85,** ***p* = 0.008**	***B* = 0.35,** ***p* = 0.002**
Mean per day *	***F*(7,388) = 2.81,** ***p* = 0.007**	*F*(1,388) = 1.90, *p* = 0.169	*F*(1,388) = 1.87, *p* = 0.172	***F*(4,388) = 3.43,** ***p* = 0.009**	*F*(1,388) = 1.26, *p* = 0.262
Use of PRN medication for aggression or anxiety **					
*Presence/absence*	**χ^2^(7) = 35.08,** ***p* < 0.001**	***B* = 0.15,** ***p* < 0.001**	*B* = −0.09, *p* = 0.549	*Wald* = 1.64, *p* = 0.801	*B* = 0.11, *p* = 0.465
Mean per admission *	***F*(7,628) = 5.73,** ***p* < 0.001**	***F*(1,628) = 26.56,** ***p* < 0.001**	***F*(1,628) = 5.97,** ***p* = 0.015**	*F*(4,628) = 1.35, *p* = 0.252	***F*(1,628) = 3.93,** ***p* = 0.048**
Use of PRN medication for sleep **					
*Presence/absence*	**χ^2^(7) = 15.79,** ***p* = 0.027**	***B* = 0.08,** ***p* = 0.014**	*B* = −0.23, *p* = 0.199	*Wald* = 0.65, *p* = 0.96	***B* = 0.50,** ***p* = 0.007**
Mean per admission *	***F*(7,787) = 5.37,** ***p* < 0.001**	***F*(1,787) = 33.13,** ***p* < 0.001**	***F*(2,787) = 4.12,** ***p* = 0.043**	*F*(4,787) = 0.41, *p* = 0.799	***F*(1,798) = 5.10,** ***p* = 0.024**
Use of PRN medication for pain **					
*Presence/absence*	**χ^2^(7) = 62.85,** ***p* < 0.001**	***B* = 0.10,** ***p* < 0.001**	***B* = −0.92,** ***p* < 0.001**	*Wald* = 7.84, *p* = 0.098	*B* = 0.22, *p* = 0.127
Mean per admission *	***F*(7,486) = 3.13,** ***p* = 0.003**	***F*(1,486) = 17.43,** ***p* < 0.001**	*F*(1,486) = 0.05, *p* = 0.829	*F*(4,486) = 0.69, *p* = 0.599	*F*(1,486) = 0.05, *p* = 0.821

Notes. * indicates that analysis includes only admission where event of interest occurred at least once. ** indicates that medication data were available for a subset of encounters (1294 encounters).

## Data Availability

Data presented in this study are available on request from the corresponding author.
